# Small Bowel Obstruction and Enterococcus Meningitis: Rare Complications of Ventriculoperitoneal Shunt Placement

**DOI:** 10.7759/cureus.40575

**Published:** 2023-06-17

**Authors:** Vincent B Gonzalez, Lisa M Pace, Matthew Johnson, Darren Klawinski

**Affiliations:** 1 Pediatrics, University of Florida College of Medicine – Jacksonville, Jacksonville, USA; 2 Pediatrics, University of Florida College of Medicine – Gainesville, Gainesville, USA; 3 Pediatric Hematology/Oncology, Nemours Children's Health System, Jacksonville, USA

**Keywords:** endoscopic third ventriculostomy, pilomyxoid astrocytoma, gram-positive meningitis, ventriculoperitoneal shunt, small-bowel obstruction

## Abstract

A ventriculoperitoneal (VP) shunt is a commonly used mechanical device indicated for congenital and acquired hydrocephalus in children. Although VP shunt failure is not uncommon, the symptomatology and cause of failure can vary. In this case, we describe intestinal obstruction in a three-year-old Caucasian female with a history of Pilomyxoid Astrocytoma and VP shunt placement for the management of hydrocephalus. Surgical exploration revealed ischemia of the terminal ileum secondary to VP shunt tubing-induced adhesions requiring bowel resection. A secondary VP shunt infection due to Enterococcus faecalis was also noted. Our case highlights a unique presentation of intestinal obstruction and infection that should serve to increase provider suspicion when evaluating patients presenting with abdominal distention and pain with presence of a VP shunt.

## Introduction

Central nervous system (CNS) tumors are the second most common type of malignancy seen in pediatric patients [1]. Pilocytic astrocytoma, a low-grade glioma typically involving the posterior fossa/infratentorial area, accounts for an estimated 15.6% of all pediatric brain tumors [2]. As these tumors are typically well-circumscribed, surgical excision remains the chosen treatment modality [3]. While complete resection is considered curative, the development of post-resection hydrocephalus occurs in an estimated 10-40% of patients [4]. For patients with both communicating and non-communicating hydrocephalus, placement of a ventriculoperitoneal (VP) shunt remains a mainstay of treatment. VP shunts function in children with hydrocephalus to divert cerebrospinal fluid (CSF) out of the CNS and into the peritoneal cavity [5].

Although VP shunt failure is not uncommon, the symptomatology and cause of failure can vary. While intrinsic catheter obstruction is the most common cause of VP shunt failure, infection is the second most common cause of failure with many cases occurring within the first few weeks after placement. Staphylococcus species predominantly cause both early and late-onset infections while enterococcus is an infrequent cause of meningitis, reportedly accounting for only 0.3-4% of cases [6]. Common abdominal complications associated with VP shunt placement include volvulus, pseudocyst formation, and distal shunt migration out of the peritoneal cavity [7]. Intestinal obstruction secondary to adhesion formation by shunt tubing is exceedingly rare [8,9]. Here we describe a unique presentation of intestinal obstruction and subsequent enterococcus infection in a three-year-old Caucasian female with a history of Pilomyxoid Astrocytoma and VP shunt placement for the management of hydrocephalus.

## Case presentation

**Figure 1:**
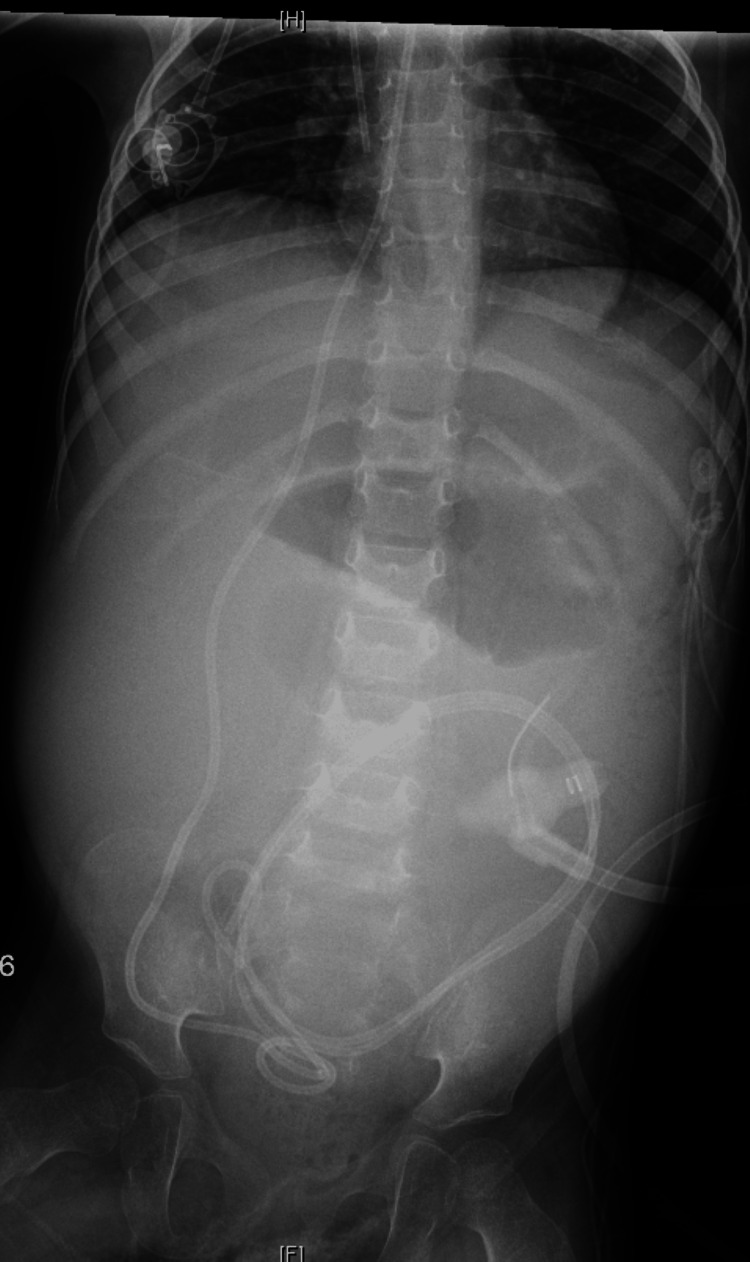
Initial abdominal X-ray revealing nonspecific bowel gas pattern with aerated loops of bowel most consistent with intestinal ileus

A three-year-old female receiving treatment for a Pilomyxoid Astrocytoma, status post tumor biopsy with partial resection and placement of a VP shunt one year prior for management of hydrocephalus, presented for weekly Vincristine/Carboplatin chemotherapy. At this time, she was noted to have significant abdominal distention with multiple episodes of emesis. History was notable for an emergency department (ED) visit two days earlier secondary to persistent non-bloody, non-bilious (NBNB) emesis. At that visit, a VP shunt series was performed and found to be normal. A blood culture was also drawn which resulted in no growth of pathogenic organisms. The patient was discharged home with plans for continued supportive care which included maintaining hydration status and use of over-the-counter analgesics/antipyretics. Following discharge, her symptoms briefly resolved but returned the night prior to our encounter. Her chemotherapy was held and an abdominal X-ray was obtained revealing intestinal ileus (Figure 1).

The patient was directly admitted to the pediatric hematology/oncology inpatient unit for further symptom management.

On physical examination, the patient was noted to have significant abdominal distention with hypoactive bowel sounds. She endorsed moderate, generalized abdominal pain to palpation. Complete blood count (CBC) obtained two days prior in the ED was unremarkable and was not repeated at the time of admission. A basic metabolic panel (BMP) was obtained and was unremarkable. Her gastrostomy tube was vented with resulting large volume bilious drainage greater than 300mL. The decision was made to insert a nasogastric Replogle, place the patient on an NPO diet, and administer a rectal enema.

**Figure 2:**
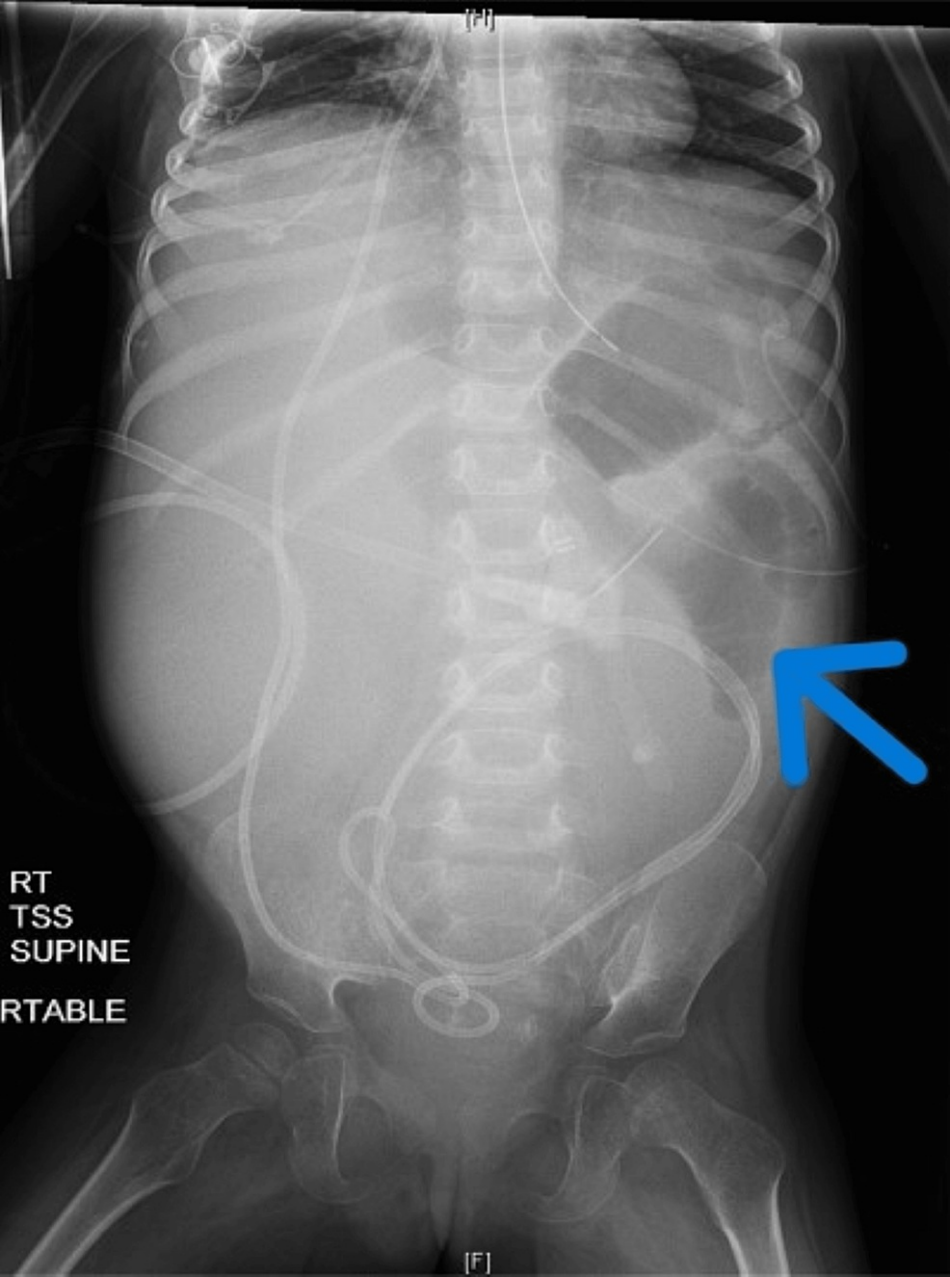
Repeat abdominal X-ray revealing persistent evidence of bowel gas paucity and bowel loop distention as identified by blue arrow

She had two small-volume bowel movements after an additional enema was administered on the second day of her hospital admission. A repeat abdominal X-ray was obtained and revealed no change from previous imaging with persistent evidence of bowel gas paucity and bowel loop distension (Figure 2).

Throughout the day the patient continued to experience intermittent worsening abdominal pain. Abdominal ultrasound was obtained to evaluate for intussusception and was found to be normal.

**Figure 3:**
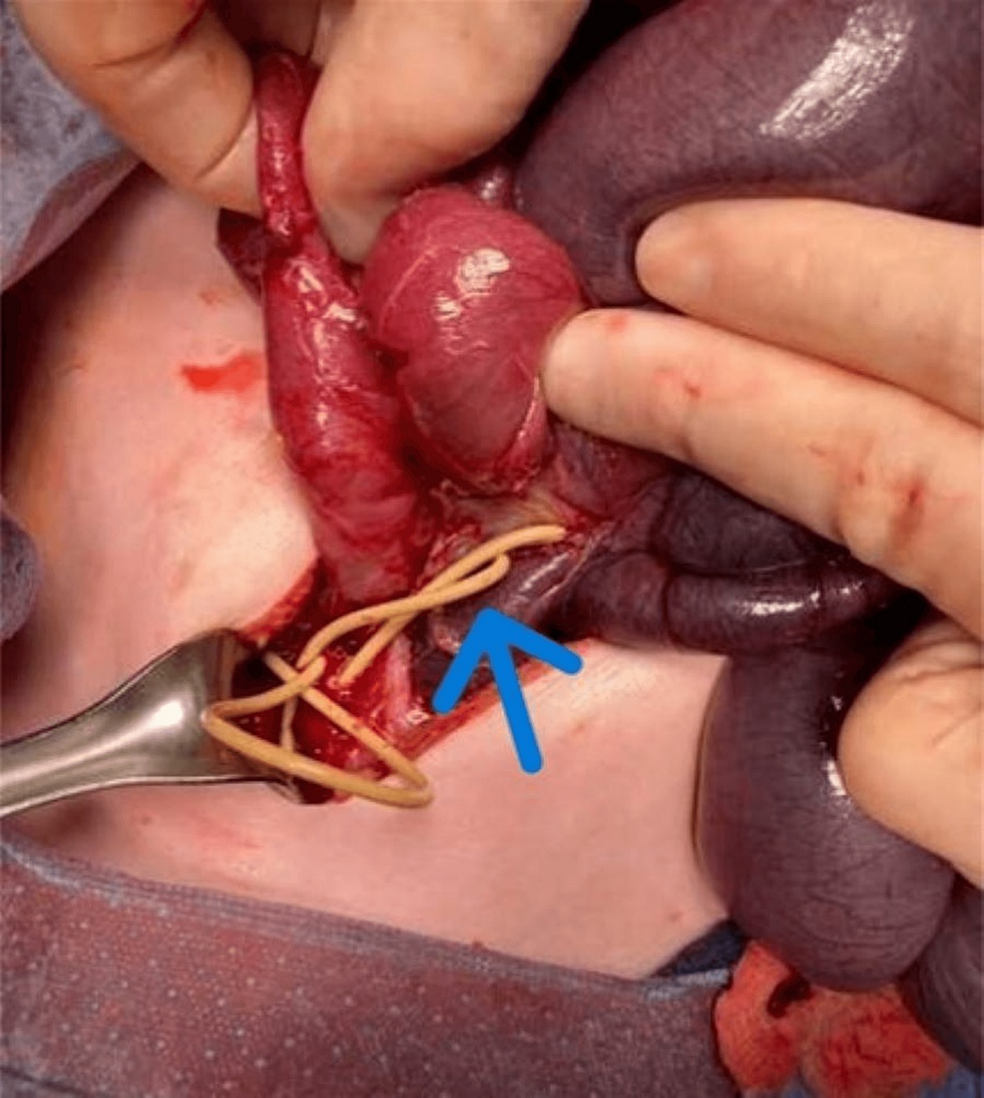
Intraoperative photograph showing a visualized area of terminal ileum with adhesive band pinching, as identified by blue arrow, and stricture development

On the morning of hospital day 3, the patient's abdominal X-ray remained unchanged and she continued to have large volume bilious output via her Replogle and gastrostomy tube. She developed tachypnea, tachycardia, and fever. Due to her progressively worsening clinical picture, she was taken by the general surgery team for exploratory laparotomy. In the operating room, the patient was noted to have significant adhesions in the pelvis and bilateral lower quadrants secondary to VP shunt tubing (Figure 3).

Her VP shunt tubing was found within adhesions as well as fibrinous pockets within her pelvis. Additionally, an area of terminal ileum was noted to be ischemic secondary to adhesion band pinching and stricture development. As a result of these findings, approximately five centimeters of small bowel was resected and an anastomosis was performed. The patient was transferred back to the hematology/oncology floor for continued postoperative management.

On postoperative day (POD) 3, she developed recurrent fevers despite antipyretic therapy. Percutaneous puncture of the VP shunt was performed and two milliliters of clear to cloudy CSF were obtained with white sediment also noted. CSF gram stain was performed and notable for gram-positive cocci identified as Enterococcus faecalis. The decision was made to proceed with complete shunt removal with insertion of bilateral external ventricular drains (EVD). Two weeks later the patient underwent re-internalization of her VP shunt system with removal of EVDs. She recovered postoperatively in the pediatric intensive care unit prior to transfer back to the hematology/oncology service. She completed a two-week course of Ampicillin for management and had no further complications.

## Discussion

The peritoneal end of a VP shunt catheter increases the patient's risk of developing a variety of intra-abdominal issues including obstruction. A literature review performed by Zhao et al. revealed shunt-related volvulus and knotting of catheter tubing to be the most common causes of mechanical obstruction in the pediatric population [8]. Intestinal blockage secondary to shunt tubing-induced adhesions, as seen in our patient, is a phenomenon rarely reported in the literature [8,9]. To the best of our knowledge, our case is unique to those previously reported in that our patient presented one year after VP shunt placement. The prolonged presence of our patient’s shunt prior to the development of intestinal obstruction should serve to increase provider awareness of potential long-term consequences of shunt placement.

VP shunt infection leading to failure has a reported incidence ranging from 5-15% [10]. Known risk factors include patient age, previous shunt failure, and duration of shunt surgery [10]. Enterococcus, a gram-positive colonizer of the gastrointestinal tract, is a rare source of infection with Enterococcus faecalis and Enterococcus faecium accounting for the majority of cases [11]. The presence of CSF devices, such as VP shunts, has been shown to predispose patients to enterococcus meningitis via unclear mechanisms [12]. Our patient developed an infection shortly after intestinal obstruction and abdominal surgery suggesting these may also place patients at risk for an ascending CNS infection.

While VP shunts are an important management strategy for patients with hydrocephalus, alternatives to VP shunt placement exist. In particular, some patients with non-communicating hydrocephalus may also be candidates for endoscopic third ventriculostomy (ETV) [13,14]. ETV is a surgical procedure in which an opening is created within the floor of the third ventricle to allow CSF to flow freely with eventual absorption by the arachnoid villi [15]. In adults, ETV has comparable therapeutic effects to VP shunt placement with fewer complications [16]. In children, ETV has been shown to have better social domain quality of life outcomes with similar resolution rates [16]. Absolute indications for ETV have yet to be defined.

## Conclusions

In this case, we report a unique complication of VP shunt placement. Overall, VP shunt malfunction and failure is not unusual, particularly in the setting of intracranial tumors. It is important to consider that these problems may occur extracranially as peritoneal adhesions, small bowel obstruction, or stricture formation. This case should increase pediatric provider awareness of potential VP shunt complications as early recognition and intervention are essential.
